# A ZIF-8-Based High-Performance Glucose Electrochemical Detection Platform Constructed Using a Multi-Layer Interface Optimization Strategy

**DOI:** 10.3390/s25227064

**Published:** 2025-11-19

**Authors:** Canjie Hu, Pengjia Qi, Lichao Liu, Yang Chen, Jijun Tong

**Affiliations:** 1 School of Information Science and Engineering (School of Cyber Science and Technology), Zhejiang Sci-Tech University, Hangzhou 310018, China; 2023220704029@mails.zstu.edu.cn; 2 Zhejiang Key Laboratory of Research and Translation for Kidney Deficiency-Stasis-Turbidity Disease, Hangzhou 310018, China; 3 College of Naval Architecture and Ocean Engineering, Naval University of Engineering, Wuhan 430033, China; 4 School of Computer Science and Technology (School of Artificial Intelligence), Zhejiang Sci-Tech University, Hangzhou 310018, China

**Keywords:** glucose detection, electrochemical biosensor, MWCNTs, ZIF-8, Gox

## Abstract

**Highlights:**

**What are the main findings?**

- A high-performance glucose biosensor was fabricated using a multilayered MWCNTs/PB/ZIF-8@GOx/CS composite.
- The sensor achieved rapid response, wide linear range, high sensitivity, ultra-low detection limit, and excellent operational stability.

**What are the implications of the main findings?**

- The developed sensor shows strong potential for real-time and accurate clinical glucose monitoring.
- This fabrication approach can be extended to construct high-performance biosensing platforms for other biomolecules.

**Abstract:**

To meet the demand for rapid and accurate glucose determination in clinical diagnostics, food testing, and related fields, this study developed a high-performance electrochemical glucose biosensor based on multi-walled carbon nanotubes/Prussian blue/zeolitic imidazolate framework-8@glucose oxidase/chitosan (MWCNTs/PB/ZIF-8@GOx/CS). The MWCNTs/PB conductive network significantly accelerated electron transfer and catalytic activity, while the ZIF-8, with its regular pore structure and high specific surface area, provides an efficient microenvironment for the immobilization and conformational stabilization of glucose oxidase (GOx), thereby improving substrate diffusion and maintaining enzyme activity. The MWCNTs/PB/ZIF-8@GOx/CS sensor demonstrates excellent sensing performance, featuring a wide linear response to glucose concentrations ranging from 4.8 μM to 2.24 mM, a high sensitivity of 579.57 μA/mM/cm^2^, and a low detection limit of 0.55 μM (S/N = 3). In addition, the sensor performs excellent repeatability (RSD = 1.49%) and retained 86.23% of its initial response after 3 weeks of storage at 4 °C, highlighting its strong potential for practical application in glucose detection.

## 1. Introduction

Glucose (Glu) is the primary energy source for living cells, but excessive intake can lead to diabetes and other health problems [1,2,3]. The World Health Organization predicts that diabetes will rank as the seventh leading cause of death worldwide by 2030 [4]. However, traditional blood glucose testing mostly employs invasive methods such as finger prick, which not only has high costs but also is highly invasive, thereby limiting the monitoring frequency for diabetic patients [5,6].

In recent years, electrochemical glucose sensing has emerged as a compelling alternative or complement to traditional methods due to its advantages of high sensitivity, low cost, portability and easy integration [7,8]. Compared with techniques such as high-performance liquid chromatography and colorimetry, electrochemical approaches are better suited to rapid quantification and online monitoring [9,10,11]. However, practical applications face the ongoing challenge of balancing high sensitivity, wide linear range and long-term stability of the sensor, to maintain catalytic performance in complex environments, remains a core issue that needs to be solved [12,13].

Glucose oxidase (GOx) is widely used in construction of glucose electrochemical sensors due to its high selectivity and good stability [14]. However, GOx is relatively fragile and easily affected by the external environment. Direct immobilization on the electrode surface often causes conformational changes, which reduces enzymatic activity and limits the stability and sensitivity of the sensor. To improve sensor performance, researchers often introduce multi-walled carbon nanotubes (MWCNTs) as conductive reinforcement materials because of their excellent electron transport properties and large specific surface area. In addition, carboxyl-modified MWCNTs introduce oxygen-containing functional groups such as carboxyl groups on their surface, which can significantly increase the effective adsorption sites and thus improve the sensitivity of the sensor [15]. Meanwhile, Prussian blue (PB), an artificial hydrogen peroxide reductase, exhibits excellent electrocatalytic activity toward hydrogen peroxide (H_2_O_2_) reduction at low potentials, thereby significantly enhancing the sensitivity and anti-interference capabilities of electrochemical detection [16,17,18]. Although MWCNTs and PB exhibit outstanding electronic properties, they offer limited capacity for robust enzyme immobilization, making it difficult to effectively maintain enzyme activity in complex or extreme environments. To address this issue, biopolymers such as chitosan (CS) have been widely employed as stabilizing and adhesive matrices that enhance enzyme retention, coating integrity, and surface hydrophilicity. CS provides abundant amino and hydroxyl groups that facilitate enzyme immobilization and improve the biocompatibility and mechanical stability of the electrode coating, making it an indispensable component in advanced biosensor architectures [19].

Metal–organic frameworks (MOFs) have attracted widespread attention in the field of biosensing due to their high specific surface area, rich pore structure, active metal sites and good biocompatibility [20,21]. Zeolite imidazolate framework-8 (ZIF-8), as a representative MOF, is composed of zinc ions and 2-methylimidazole (2-MIM) through tetrahedral coordination. It has a highly porous structure and excellent chemical and thermal stability. Its mild, facile synthesis has enabled broad use in electrochemical biosensors and in protective encapsulation of biomacromolecules [22]. The pore structure of ZIF-8 not only provides a high specific surface area, but also helps preserve enzyme conformation, thereby improving activity retention under extreme conditions. Therefore, it is regarded as an ideal enzyme or nanozyme immobilization carrier [23,24]. Enzyme immobilization methods in MOF-based supports can be primarily categorized into two methods: post-synthetic adsorption and in situ co-precipitation [25]. The former involves synthesizing the MOF framework and then introducing the enzyme molecules via impregnation and adsorption. This method has been successfully used to immobilize enzymes such as GOx and horseradish peroxidase (HRP), demonstrating excellent catalytic activity and reusability [26,27]. However, this method is cumbersome, time-consuming, and has limitations in pore size matching. In contrast, the in situ co-precipitation method is simpler and milder: simply dissolving a metal ion (such as Zn^2+^), an organic ligand (such as 2-methylimidazole), and the enzyme in aqueous solution rapidly forms an enzyme@ZIF-8 complex. Numerous experimental studies have demonstrated that this method can efficiently encapsulate GOx/HRP at room temperature, achieving good encapsulation efficiency and structural compactness [28]. In addition, ZIF-8-Enzyme complexes based on in situ embedding or post-synthetic impregnation are significantly superior to free enzymes in terms of thermal stability, pH tolerance, organic solvent tolerance and long-term storage stability. For example, Yan et al. reported a Pt-GOx@H-ZIF-8 core–shell architecture, and Lin et al. developed a GOx@ZIF-8-based bio-sensor, both showing higher retention of enzyme catalytic activity and improved enzyme structural stability under high temperature, extreme pH, and organic-phase conditions [29,30].

Building on these advances, this study designed and constructed an electrochemical glucose sensor based on a MWCNT/PB/ZIF-8@GOx/CS composite. In this configuration, the CS layer acts as a biocompatible polymer matrix that facilitates enzyme immobilization, preserves enzymatic activity, and enhances interfacial stability. Notably, multicomponent synergistic regulation has been demonstrated as an effective strategy for improving catalytic kinetics and selectivity in the electrocatalysis field [31]. Guided by this strategy, our multilayer composite integrates the excellent conductivity of MWCNTs (promoting rapid electron transfer), the efficient H_2_O_2_ catalysis of PB (strengthening H_2_O_2_ reduction for signal amplification), and the enzyme encapsulation and protection capability of ZIF-8, resulting in synergistic improvements in both sensitivity and stability. These results provide a promising and strategy-supported approach for the development of high-performance electrochemical biosensors.

## 2. Experimental Section

### 2.1. Materials

Multi-walled carbon nanotubes (MWCNTs, AR ≥ 99.5%; Shenzhen Suiheng Graphene Technology Co., Ltd., Shenzhen, China) were used as received. Zinc nitrate hexahydrate (Zn(NO_3_)_2_·6H_2_O, 0.1 M; Fulin Standard Titration Solution, Shenzhen, China) was employed as the zinc source. 2-Methylimidazole (2-MIM, AR ≥ 99.5%), bovine serum albumin (BSA, AR ≥ 99%), glucose oxidase (GOx, 100 units/mg), hydrochloric acid (HCl, 37%), and chitosan (CS, deacetylation degree ≥85%) were purchased from McLean Reagent Enterprise Store (Shanghai, China). Ferric chloride (FeCl_3_), sodium chloride (NaCl), potassium chloride (KCl), potassium ferricyanide (K_3_[Fe(CN)_6_]), sodium dihydrogen phosphate (NaH_2_PO_4_), disodium hydrogen phosphate (Na_2_HPO_4_), lactic acid (LA), uric acid (UA), and ascorbic acid (AA) were all analytically pure and purchased from Aladdin Reagent Co., Ltd. (Shanghai, China). Screen-printed carbon electrodes (SPCEs) were purchased from Shenzhen Haoyang Technology Co., Ltd. (Shenzhen, China). All experimental water was deionized.

### 2.2. Equipment

The main instruments used in the experiments included an electrochemical workstation (DH7000D, Jiangsu Donghua, Taizhou, China), a scanning electron microscope (SEM, GeminiSEM500, Zeiss, Oberkochen, Germany), a Fourier transform infrared spectrometer (FT-IR, Nicolet iS10, Thermo Fisher Scientific, Waltham, MA, USA), an X-ray photoelectron spectrometer (XPS, ESCALAB Xi, Thermo Fisher Scientific, Waltham, MA, USA), and an X-ray diffractometer (XRD, A8 Advance, Bruker, Ettlingen, Germany). All measurements were performed at 20 ± 2 °C.

### 2.3. Preparation of Sensor Electrodes

Preparation of ZIF-8@GOx: ZIF-8 was synthesized according to the previous method [30]. By adjusting the molar ratio of Zn^2+^ to 2-methylimidazole (2-MIM), glucose oxidase (GOx) and bovine serum albumin (BSA) were simultaneously introduced to achieve enzyme encapsulation. 4.0 mL of 0.1 mol/L Zn(NO_3_)_2_·6H_2_O solution (containing 0.4 mmol Zn^2+^) was added to the centrifuge tube, followed by the sequential addition of 25 mg of GOx and 10 mg of BSA. The solution was made up to 5 mL with deionized water and left at room temperature for 10 min to promote the complexation of GOx and BSA with Zn^2+^. Another 985 mg of 2-MIM (12 mmol) was dissolved in 5.0 mL of deionized water and quickly added to the above solution, and stirred for 5 min. During the reaction, the solution gradually changed from transparent yellow to turbid, and was left to stand overnight to promote crystallization. The supernatant was discarded, and a light yellow powder was obtained after centrifugation. The precipitate was recovered by centrifugation, and washed three times with deionized water to remove unbound GOx, BSA and Zn^2+^. Finally, the ZIF-8@GOx material was obtained.

Fabrication of MWCNTs sensor: MWCNTs were dispersed in ethanol and a uniform suspension was obtained after 30 min of ultrasonic treatment. Solutions of increasing concentrations (1.0, 1.5, and 2.0 mg/mL) were prepared, respectively. Then, 4 μL of each solution was dropped onto the clean electrode surface, and the samples were dried at room temperature to form modified layers. The electrodes prepared with different MWCNT concentrations were designated as MWCNTs1.0, MWCNTs1.5, and MWCNTs2.0.

Fabrication of MWCNTs/PB sensor: The electrodes that have been modified with different concentrations of MWCNTs were, respectively, immersed in the prepared PB solution (2.5 mM FeCl_3_, 2.5 mM K_3_[Fe (CN)_6_], 0.1 M KCl, 0.1 M HCl). The CV scans were performed within the potential range of −0.2 to +0.6 V, with a potential step (Estep) of 1 mV and a scanning rate of 50 mV/s, for 10, 15 and 20 cycles respectively. Thus, the electrodes labeled as MWCNTs1.0/PB10, MWCNTs1.0/PB15, MWCNTs1.0/PB20, MWCNTs1.5/PB10, MWCNTs1.5/PB15, MWCNTs1.5/PB20, MWCNTs2.0/PB10, MWCNTs2.0/PB15, and MWCNTs2.0/PB20 were obtained.

**Figure 1:**
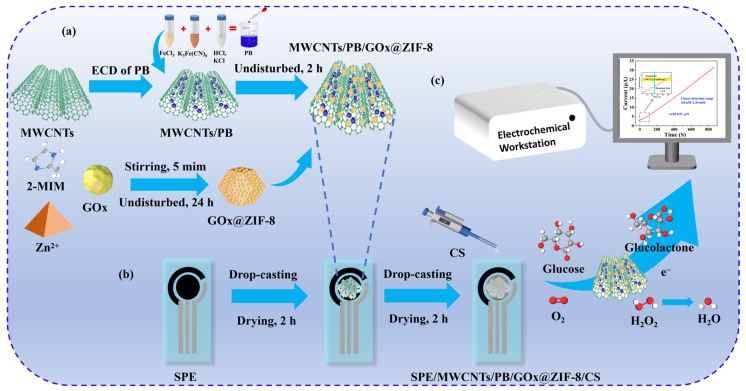
(**a**) Preparation process of the MWCNTs/PB/ZIF-8@GOx composite. (**b**) Flowchart illustrating the fabrication of the MWCNTs/PB/ZIF-8@GOx/CS modified sensor. (**c**) Schematic illustration of the glucose detection mechanism.

Preparation of MWCNTs/PB/ZIF-8@GOx/CS sensor: 50 μL of ZIF-8@GOx composite material was dispersed in 120 μL of deionized water, thoroughly mixed, and then applied onto the electrode surface that had been modified with MWCNTs/PB. The mixture was left to dry naturally at room temperature. After drying, 2 μL of 1% (*w*/*v*) CS solution was dropped onto the electrode surface to form a protective layer, thereby constructing a stable MWCNTs/PB/ZIF-8@GOx/CS sensor. Figure 1 shows the schematic diagram of the fabrication of the MWCNTs/PB/ZIF-8@GOx/CS sensor.

## 3. Results and Discussion

### 3.1. Morphological and Structural Characterization

**Figure 2:**
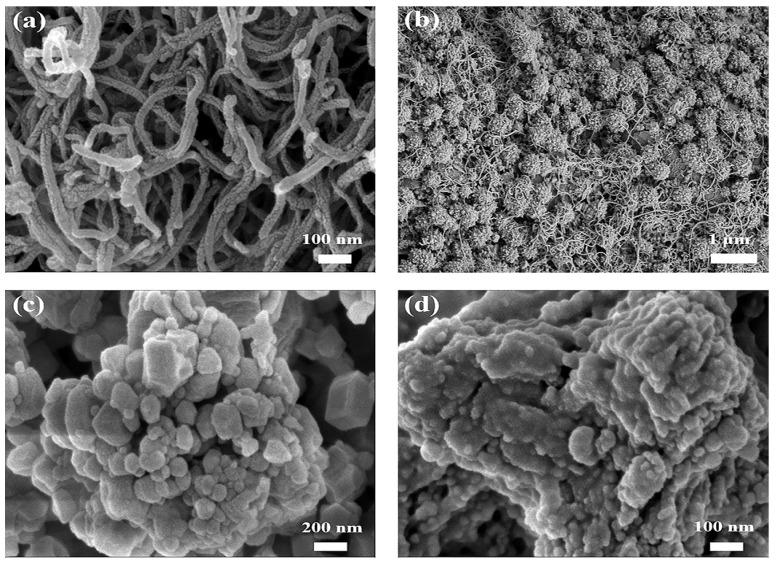
SEM images of electrode modification materials. (**a**) MWCNTs. (**b**) PB nanoparticles uniformly deposited on the surface of MWCNTs. (**c**) ZIF-8. (**d**) ZIF-8@GOx composite.

To verify the morphological characteristics and particle distribution changes of the ZIF-8@GOx composite material, Figure 2 presents the SEM morphologies of MWCNTs, PB, ZIF-8, and ZIF-8@GOx. As shown in Figure 2a, the MWCNT-modified layer consists of entangled tubular structures with diameters of tens of nanometers, forming a compact three-dimensional conductive network that markedly increases the electrode’s specific surface area and electron transfer efficiency. Figure 2b shows that PB particles are uniformly deposited on the MWCNTs surface in spherical or aggregated forms, with particle sizes ranging from tens to hundreds of nanometers, forming a rough surface that enhances electrocatalytic activity. The synergistic layered structure thus integrates both conductivity and catalytic functionality. As shown in Figure 2c, the synthesized ZIF-8 exhibits a typical dodecahedral morphology, with smooth surfaces and an average particle diameter of approximately 450 nm. These structural characteristics agree well with previous reports, confirming the successful formation of the crystal framework [30,32]. In contrast, the ZIF-8@GOx particles in Figure 2d exhibit rougher surfaces, indistinct edges, and slight local aggregation and shrinkage. These changes may be attributed to the involvement of GOx in the nucleation and growth processes, which disrupts the orderly coordination of metal ions and ligands, thereby affecting the crystal morphology and size uniformity. Combined with the changes in morphological characteristics and particle size distribution, it can be inferred that GOx has been successfully embedded in the ZIF-8 framework, the composite structure is stable, and the overall crystal morphology is well maintained, confirming the successful synthesis of the ZIF-8@GOx composite material.

**Figure 3:**
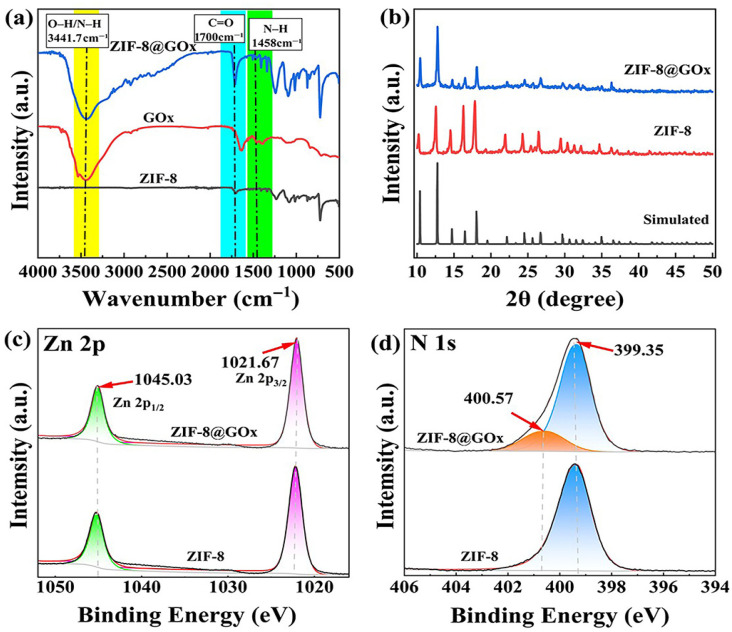
Structural and chemical characterization of ZIF-8@GOx composites. without BSA (**a**) FT-IR spectrum. (**b**) XRD pattern. (**c**) XPS Zn 2p. (**d**) XPS N 1 s.

FT-IR spectroscopy results are shown in Figure 3a, where GOx exhibits characteristic absorption peaks at 3441.7 cm^−1^, 1655.2 cm^−1^, and 1458 cm^−1^, corresponding to the stretching vibrations of hydroxyl and amino groups, the carbonyl stretching vibration of amide I, and the bending vibration of the amino group, respectively. The ZIF-8@GOx composite retains these characteristic peaks, suggesting that the enzyme preserves its structural integrity during the encapsulation process. The absorption band at 3431.5 cm^−1^ becomes broader, likely due to alterations in the hydrogen-bonding environment. Furthermore, the absorption peak of the amide I band in ZIF-8@GOx shifts to approximately 1700 cm^−1^, representing a blue shift relative to pure GOx. This is presumably due to coordination or hydrogen bonding interactions between the C=O groups and metal ions or imidazole ligands, reflecting changes in the enzyme’s conformation and local microenvironment, further confirming successful incorporation of the enzyme into the ZIF-8 framework.

The XRD pattern presented in Figure 3b demonstrates that the diffraction peaks of ZIF-8@GOx are nearly identical to those of pure ZIF-8 and its simulated spectrum, without the appearance of new phases or significant peak shifts. This indicates that enzyme incorporation does not compromise the crystallinity of the framework, and the ZIF-8 structure remained intact and ordered.

XPS analysis further supports this conclusion. The Zn 2p spectrum illustrated in Figure 3c exhibits characteristic Zn 2p3/2 and Zn 2p1/2 peaks at 1021.35 eV and 1044.55 eV, respectively, demonstrating a typical spin–orbit splitting structure. Additionally, the N 1 s spectrum shown in Figure 3d reveals a single peak at 399.35 eV for the ZIF-8 sample, corresponding to coordinated nitrogen (Zn–N) in the ligand. In contrast, the ZIF-8@GOx sample exhibits an additional shoulder peak at 400.57 eV, which is attributed to the amide or amino nitrogen in the enzyme, thereby validating the successful encapsulation of GOx within the framework.

### 3.2. Interfacial Electron Transfer Properties

**Figure 4:**
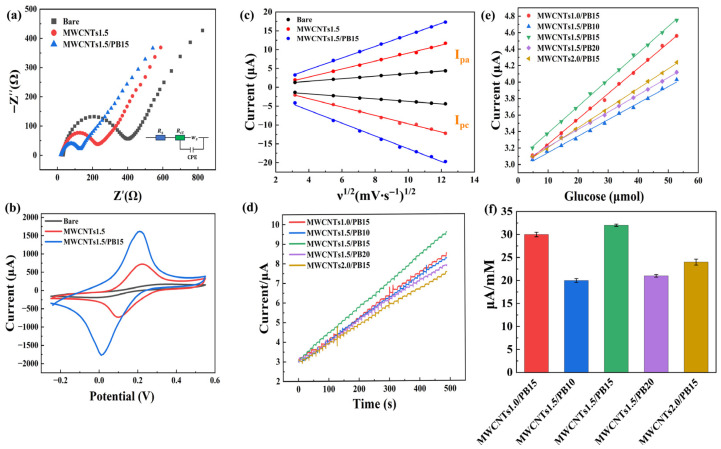
(**a**) Nyquist plots (EIS) of various electrodes recorded in 5.0 mM [Fe(CN)_6_]^3−^/^4−^ and 0.1 M KCl at room temperature (frequency range: 0.1 Hz–100 kHz; AC amplitude: 5 mV), with the equivalent circuit model. (**b**) CV curves of different modified electrodes recorded at 50 mV/s within −0.2–0.6 V (vs. Ag/AgCl) in 5.0 mM [Fe(CN)_6_]^3−^ and 0.1 M KCl at room temperature. (**c**) Linear relationship between peak current (Ip) and square root of scan rate (ν^1^ᐟ^2^), obtained from CV measurements conducted at 10–150 mV·s^−1^. (**d**) IT response curves obtained at 0 V under different modification conditions using the MWCNTs/PB/GOx/CS sensor. (**e**) Linear fitting of response current versus glucose concentration for the sensors shown in (**d**), shown as mean ± SD (*n* = 3). (**f**) Comparison of sensitivities of various MWCNTs/PB/GOx/CS sensors, presented as mean ± SD (*n* = 3).

To systematically evaluate the effects of different modifying materials on the interfacial electron transport properties of electrodes, various electrodes were tested by electrochemical impedance spectroscopy (EIS) in a mixed solution of 5.0 mM [Fe(CN)_6_]^3−/4−^ and 0.1 M KCl. Figure 4a shows the Nyquist plots and equivalent circuit of each electrode at room temperature, where $R_{s}$ represents the solution resistance, $R_{ct}$ represents the electron transfer resistance, $W_{s}$ represents the Warburg impedance, and constant phase element (CPE) represents the capacitance. All electrodes display a semicircle in the high-frequency region and a linear tail in the low-frequency region, reflecting the synergistic effect of charge transfer and diffusion processes. The bare electrode exhibits the largest semicircle diameter and the highest corresponding $R_{ct}$, suggesting poor interfacial electron transport efficiency. Modification with MWCNTs significantly reduces the semicircle diameter and significantly decreases the $R_{ct}$, attributed to the enhanced electron transport capacity of carbon nanotubes due to their excellent conductivity and high specific surface area. Further introduction of PB further minimizes the $R_{ct}$, highlighting the synergistic effect of the MWCNTs/PB composite in constructing conductive pathways and providing charge transfer sites.

The electrocatalytic performance of each electrode was further investigated using CV. As shown in Figure 4b, the MWCNTs/PB-modified electrode exhibited the highest redox peak current, indicating superior electrochemical activity. This result is consistent with the $R_{ct}$ trend observed in the EIS analysis, further validating the enhanced electrode performance of the composite material.

The electroactive surface area (ECSA) of the electrode was calculated using the Randles-Sevcik equation:

(1) $$I_{p}\;=\;0.4463nFA_{real}C\sqrt{\frac{nFDv}{RT}}$$

where $A_{real}$ is the effective active area of the electrode, D is the corresponding diffusion coefficient (7.6 × 10^−6^ cm^2^/s), C is the concentration of the iron standard solution (Fe(CN)_6_]^3−/4−^), and v is the scan rate (50 mV/s). R is the gas constant (8.314 J/(mol·K) and T is the absolute temperature (298 K). The results showed that the ECSA of the bare electrode, MWCNT-modified electrode, and MWCNTs/PB composite-modified electrode were 0.102 cm^2^, 0.434 cm^2^ and 0.964 cm^2^, respectively. The ECSA of the composite electrode was significantly larger, which was consistent with its higher redox peak current and lower charge transfer resistance ($R_{ct}$).

**Figure A1:**
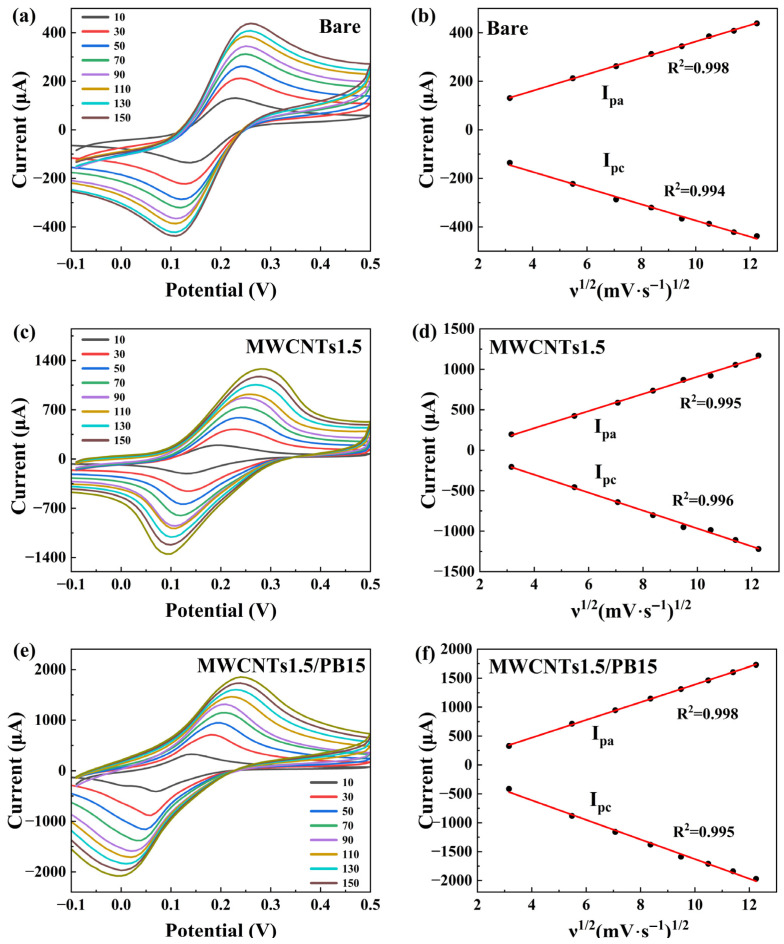
At room temperature, CV curves for different modified electrodes were recorded in 5.0 mM [Fe(CN)_6_]^3−^ and 0.1 M KCl solutions at scan rates of 10–150 mV/s within a potential range of −0.1–0.5 V (vs. Ag/AgCl) (**a**) Bare electrode, (**c**) MWCNTs1.5 modified electrode, and (**e**) MWCNTs1.5/PB15 modified electrode, showing CVs recorded at different scan rates; (**b**,**d**,**f**) corresponding linear plots of peak current ($I_{p}$) versus square root of scan rate (ν^1^ᐟ^2^), respectively.

The CV curves obtained at varied scan rates and the corresponding plots of peak current ($I_{p}$) versus the square root of the scan rate (ν^1/2^) for each electrode are provided in Figure A1 in Appendix A. Furthermore, Figure 4c summarizes the linear relationships between $I_{p}$ and ν^1/2^ for different electrodes, enabling direct comparison of their diffusion-controlled behavior. The results showed that all modified electrodes exhibited excellent linearity (R^2^ > 0.99), indicating that the electrochemical reaction was primarily diffusion-controlled. Among them, the fitting slope of the MWCNTs/PB composite-modified electrode was the largest. Combined with a larger effective electroactive area, higher redox peak current and the lowest $R_{ct}$, this further confirms that this composite structure has significant advantages in enhancing interface electron transfer and material diffusion.

### 3.3. Optimization of Electrode Modification Parameters

**Figure A2:**
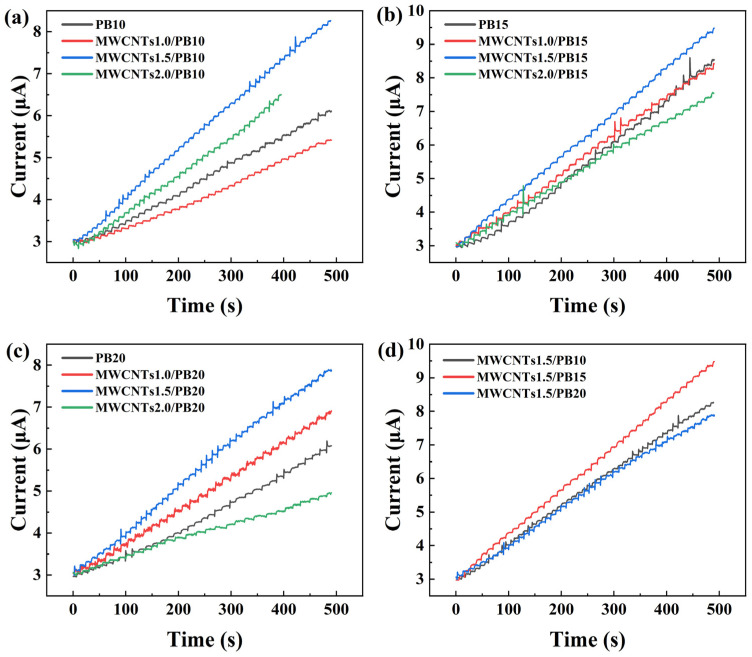
IT responses of MWCNTs/PB-modified electrodes with varying MWCNT loadings (0 mg/mL, 1.0 mg/mL, 1.5 mg/mL, and 2.0 mg/mL) and PB deposition cycles: (**a**–**c**) IT curves corresponding to PB10, PB15, and PB20 deposition cycles, respectively, illustrating the synergistic effect of MWCNT content and PB film thickness; (**d**) Focused comparison of top-performing electrode configurations screened from (**a**–**c**) (MWCNTs1.5/PB10, MWCNTs1.5/PB15, and MWCNTs1.5/PB20). Among them, MWCNTs1.5/PB15 exhibits the steepest current-time slope, confirming its status as the optimal electrode design. Glucose was injected into 100 mL PBS (0.1 M, pH 7.4) every ~10 s with each injection introducing 48 μmol of glucose.

**Figure A3:**
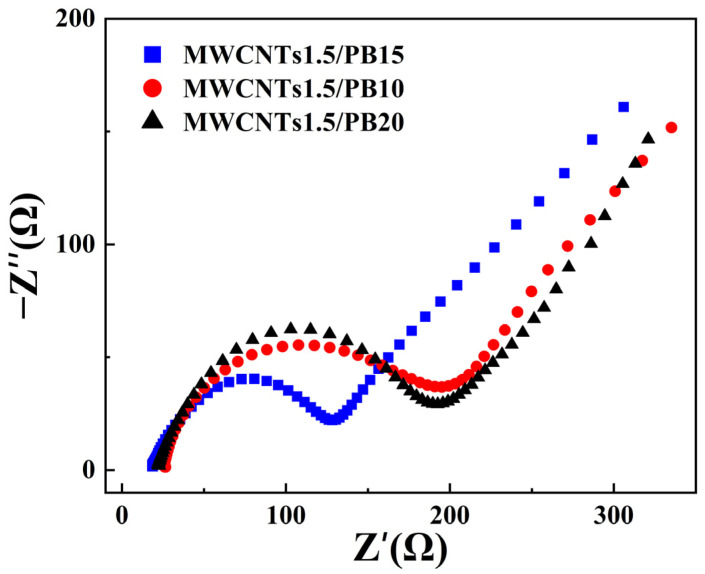
Nyquist plots (EIS) of different PB coverage cycles (10 to 20 cycles) recorded in 5.0 mM [Fe(CN)_6_]^3−^/^4−^ and 0.1 M KCl at room temperature (frequency range: 0.1 Hz–10 kHz; AC amplitude: 5 mV), with the equivalent circuit model.

Representative chronoamperometry (IT) response curves, linear fits of response current versus glucose concentration, and sensitivity comparisons of selected configurations are shown in Figure 4d–f. The complete IT response curves for all other electrode configurations and the EIS under different PB coverage cycles (10 to 20 cycles) are shown in Figure A2 and Figure A3 in Appendix A. Results revealed that increasing the MWCNTs concentration from 1.0 to 1.5 mg/mL markedly enhanced the response current, indicating improved electronic conductivity and a higher density of electroactive sites. However, a further increase to 2.0 mg/mL decreased the response, likely due to the formation of an excessively thick MWCNT network that limited effective electron transfer pathways despite the intrinsic conductivity of MWCNTs. At a MWCNT concentration of 1.5 mg/mL, as the number of PB deposition cycles increased, the mass of PB increased, and the electrocatalytic activity of the electrode gradually improved. The best performance was achieved at 15 cycles. Further increasing to 20 cycles led to a decrease in response, which was speculated to be due to the excessive thickness of the PB layer, resulting in an increase in interface impedance and a decrease in electron transfer efficiency. The comprehensive analysis indicates that the MWCNTs1.5/PB15 modified electrode can achieve a good balance between electron transfer and electrocatalytic activity, and exhibits the highest sensitivity for glucose detection. Therefore, the MWCNTs1.5/PB15 modified electrode was selected for the subsequent experiments.

### 3.4. Amperometric Glucose Sensing Performance

**Figure 5:**
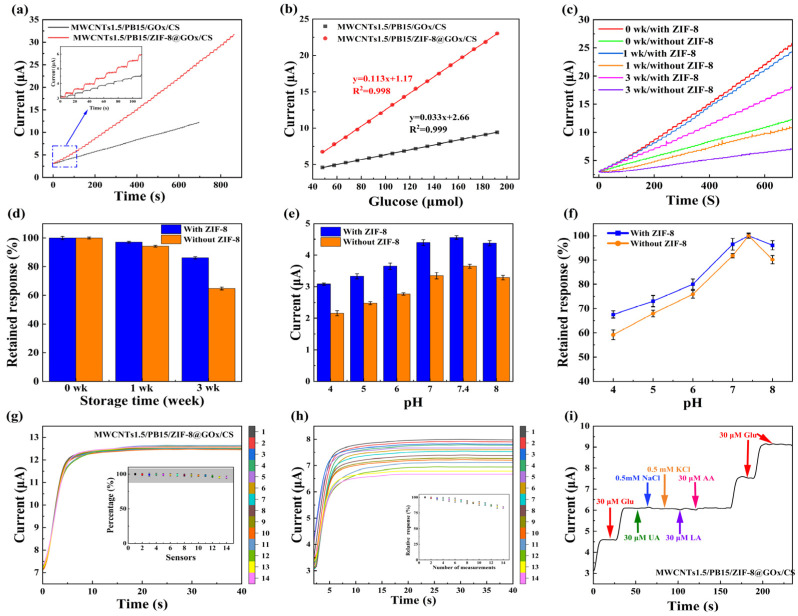
Electrochemical performance of the MWCNTs1.5/PB15/ZIF-8@GOx/CS sensor: (**a**) Ampere response of MWCNTs1.5/PB15/ZIF-8@GOx/CS electrode and control electrode after adding 48 μmol glucose to 100 mL PBS buffer (0.1 M, pH 7.4) every 10 s. (**b**) Corresponding calibration curves and linear fittings, shown as mean ± SD (*n* = 3). (**c**) Comparison of amperometric responses after 0, 1, and 3 weeks of storage (4 °C) for electrodes with and without ZIF-8. (**d**) Retained response at 0, 1, and 3 weeks. (**e**) Steady-state current responses measured at different pH values (4–8). (**f**) Normalized retained response across the pH range. (**g**) Inter-sensor reproducibility evaluated using 14 independently fabricated electrodes. (**h**) Intra-sensor repeatability over 14 consecutive measurements on the same electrode. (**i**) Selectivity test against common interferents (AA, UA, LA, NaCl, KCl) at 0 V.

Furthermore, we introduced ZIF-8 as the carrier for the immobilization of glucose oxidase, and constructed the MWCNTs1.5/PB15/ZIF-8@GOx/CS sensor, and made the MWCNTs1.5/PB15/GOx/CS sensor as a control. IT measurements were performed at different glucose concentrations and compared with a control electrode without ZIF-8, as illustrated in Figure 5a.

**Figure A4:**
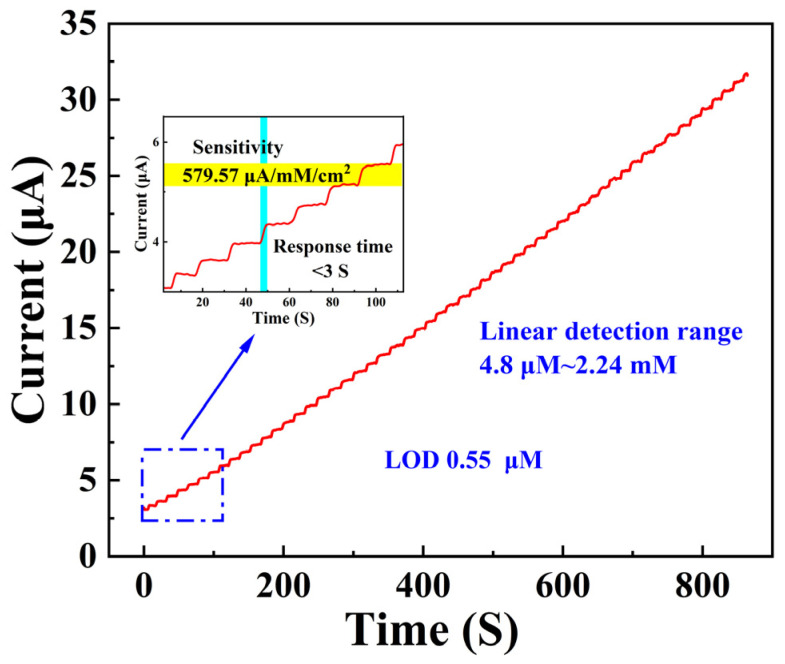
IT response of the MWCNTs1.5/PB15/ZIF-8@GOx/CS modified electrode upon successive glucose additions, where glucose was injected into 100 mL PBS (0.1 M, pH 7.4) every ~10 s with each injection introducing 48 μmol of glucose.

The enzyme-immobilized electrode exhibited significantly higher steady-state currents than the control at the same glucose concentration, indicating that the porous ZIF-8 framework not only provides a favorable microenvironment for GOx to retain its catalytic activity but also facilitates substrate diffusion and enhances the overall electron transfer efficiency of the composite system through improved enzyme dispersion and interfacial contact with the conductive matrix. As shown in Figure 5b, the biosensor displayed an excellent linear correlation between current response and glucose concentration in the range of 4.8 μM–2.24 mM (R^2^ > 0.99), with a much steeper slope than that of the control electrode. The sensitivity of the MWCNTs1.5/PB15/ZIF-8@GOx/CS sensor reached 579.57 μA/mM/cm^2^ (based on an electrode area of 0.2 cm^2^), which was approximately 70% higher than that of the MWCNTs1.5/PB15/GOx/CS sensor, with a detection limit of 0.55 μM (S/N = 3). In addition, the biosensor exhibited a rapid response time of <3 s, as presented in Figure A4 in Appendix A highlighting its potential for fast and reliable glucose monitoring.

### 3.5. Stability and Selectivity Evaluation

To ensure reliable analytical performance, we systematically evaluated long-term stability, pH stability, selectivity, reproducibility, and recurrence. Long-term stability was assessed by sealing the electrode and storing it at 4 °C, followed by amperometric measurements in 0.1 M PBS. As shown in Figure 5c,d, the response of the MWCNTs/PB/ZIF-8@GOx/CS sensor decreased only slightly over time, retaining 97.04% and 86.23% of the initial current after 1 and 3 weeks, respectively. In contrast, the control electrode, MWCNTs1.5/PB15/GOx/CS, showed a more significant decrease in response, retaining 94.30% after 1 week and only 64.72% after 3 weeks. These results indicate that ZIF-8 encapsulation significantly improves the long-term preservation of enzyme activity, which is attributed to the rigid and porous framework structure of ZIF-8 limiting conformational distortion of GOx and protecting its active conformation from environmental stress.

The pH stability of the electrode was further investigated in a 0.1 M PBS buffer solution containing 30 µM glucose in the pH range of 4–8. As shown in Figure 5e, both sensors exhibited maximum steady-state current at pH 7.4. To facilitate comparison, the currents at each pH were normalized to their respective responses at pH 7.4 (Figure 5f). The MWCNTs1.5/PB15/ZIF-8@GOx/CS sensor maintained a higher fraction of its optimal response across the pH range, confirming enhanced tolerance to deviations from physiological pH.

Furthermore, reproducibility and repeatability were rigorously evaluated. The repeatability of the glucose sensor was examined by recording the responses of 14 sensors to glucose in PBS (pH = 7.4) containing 0.1 mM glucose. As shown in Figure 5g, the relative standard deviation (RSD) of the response current detected by the glucose sensor was only 1.49%, indicating that the preparation process was controllable, the signal output was stable, and the batch-to-batch variation was minimal. As shown in Figure 5h, after 14 repeated tests, the IT curve of the sensor showed little change, and the inset shows the degree of decay of the current response after 14 sensing experiments. The current value in the 14th test remained at approximately 79.91% of the initial current value. The results indicate that the glucose biosensor modified with MWCNTs/PB/ZIF-8@GOx/CS exhibits good repeatability.

Finally, the selectivity of the biosensor was investigated, and the results are shown in Figure 5i. Common interferents such as 30 μM AA, 30 μM UA, 30 μM LA, 0.5 mM NaCl, and 0.5 mM KCl were sequentially added to the detection system. The results showed that these interferents caused negligible current changes, while each addition of 30 μM glucose produced a significant increase in the steady-state response. This demonstrates that the sensor is highly selective for glucose and effectively resists interference from common electroactive substances and inorganic salts, ensuring accurate detection in complex biological environments.

In summary, the introduction of ZIF-8 significantly improves storage stability, pH tolerance, and selectivity. Simultaneously, this preparation method ensures excellent batch-to-batch reproducibility and good internal repeatability of the sensor.

### 3.6. Comparative Analysis and Catalytic Mechanism

**Figure 6:**
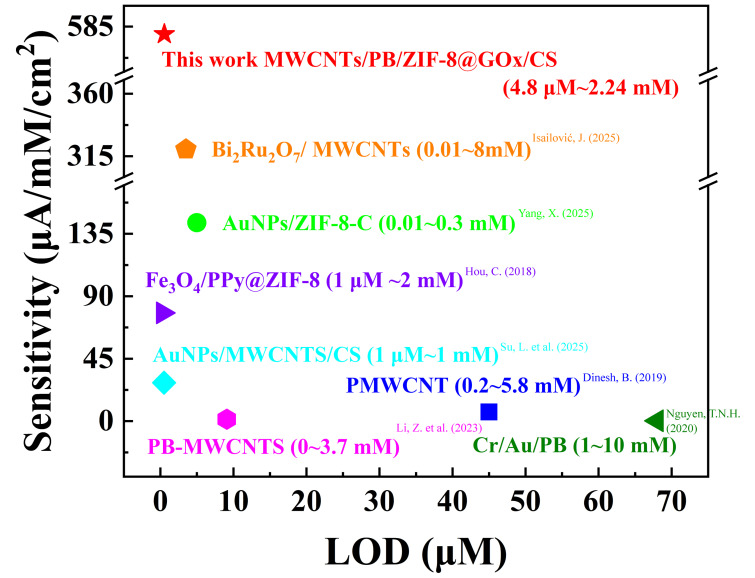
**Table 1** shows a performance comparison of some similar glucose sensors [17,31,33,36,37,38,39].

Compared with previously reported glucose sensors, as shown in Figure 6, most devices still exhibit limitations such as low sensitivity, narrow linear detection ranges, or high limits of detection (LOD). In contrast, the MWCNTs1.5/PB15/ZIF-8@GOx/CS sensor developed in this study achieves markedly superior performance in terms of sensitivity, LOD, and linear range. This improvement arises from the synergistic electron transport and catalytic effects of MWCNTs and PB, together with the precise optimization of MWCNTs concentration (1.5 mg/mL) and PB film thickness (15 electrodeposition cycles), which collectively construct an optimal interface structure with both high conductivity and stability. Moreover, the rigid porous framework of ZIF-8 provides a stable microenvironment for GOx immobilization, preserving the enzyme’s conformation and catalytic activity while facilitating substrate diffusion and product migration, thereby greatly enhancing the sensor’s low-concentration detection capability. Comparative evaluation with previously reported sensors (Table 1) further confirms the strong competitiveness of the proposed system.

**Table 1:** Performance comparison of similar glucose sensors.

Electrode	Linear Range (mM)	Sensitivity (μA/mM/cm^2^)	LOD (μM)	Operational Potential	Ref.
Cr/Au/PB	1–10	0.23	68	−0.1 v vs. Ag/AgCl	[33]
PB-MWCNTs	0–3.7	1.34	9.1	0.03 v vs. Ag/AgCl	[17]
AuNPs/ MWCNTs/CS	0.001–1	27.7	0.5	0.20 v vs. Ag/AgCl	[19]
Ti_3_C_2_Tx/ZIF-67	0.005–7.5	0.379	0.66	0.35 v vs. Ag/AgCl	[34]
Ag@TiO_2_@ ZIF-67	0.048–1	788	0.99	0.4 v vs. Ag/AgCl	[35]
AuNPs/ZIF-8-C	0.01–0.3	143	4.99	0.8 v vs. Ag/AgCl	[36]
LIG/CA/PU/ ZIF-8	0.62–20	1.778	160	0.45 v vs. Ag/AgCl	[22]
Fe_3_O_4_/PPy@ ZIF-8	0.001–2	78	0.333	0.6 v vs. Ag/AgCl	[37]
PMWCNT	0.2–5.8	6.6	45	/	[38]
Bi_2_Ru_2_O_7_/MWCNTs	0.01–8	319.88	3.5	/	[39]
MWCNTs/PB/ ZIF-8@GOx/CS	0.0048–2.24	579.57	0.55	0 v vs. Ag/AgCl	This work

**Figure 7:**
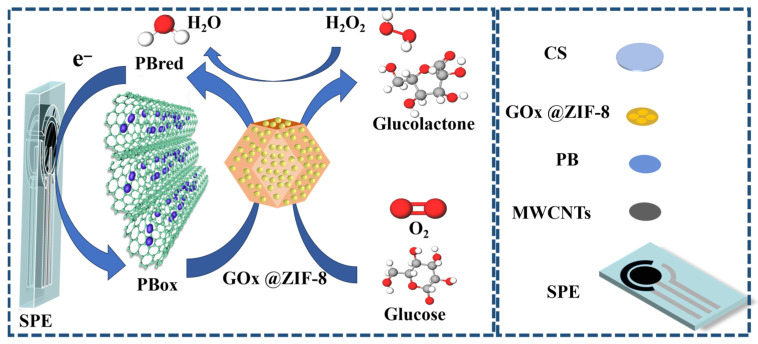
The catalytic mechanism of the MWCNTs/PB/ZIF-8@GOx/CS sensor.

The catalytic mechanism of the MWCNTs/PB/ZIF-8@GOx/CS sensor is illustrated in Figure 7. In the presence of oxygen, GOx immobilized within the ZIF-8 matrix catalyzes the oxidation of glucose to gluconolactone, accompanied by the reduction in its flavin adenine dinucleotide (FAD) cofactor to FADH_2_ (Equation (2)). The reduced cofactor subsequently transfers electrons to molecular oxygen, generating hydrogen peroxide (Equation (3)). The resulting H_2_O_2_ is electrocatalytically reduced at the Prussian Blue (PB) layer, where the reversible redox transition between oxidized PB (${PB}_{ox}$) and reduced PB (${PB}_{red}$) facilitates electron transfer to the electrode, thereby producing a measurable current response (Equation (4)).

(2) $$Glucose+GOx\left(FAD\right)\to Gluconolactone+GOx\left(FADH_{2}\right)$$

(3) $$GOx\left(FADH_{2}\right)+O_{2}\to GOx\left(FAD\right)+{{\;H}_{2}O}_{2}$$

(4) $${H_{2}O}_{2}+2{PB}_{red}\;\to \;2{PB}_{ox}+2{OH}^{-}+2e^{-}$$

## 4. Conclusions

This study presents a straightforward and efficient strategy for constructing a multilayer composite glucose electrochemical biosensor (MWCNTs/PB/ZIF-8@GOx/CS) with significantly improved performance. The incorporation of MWCNTs and PB forms a conductive electron-transfer network and an active catalytic interface, while ZIF-8 acts as a robust enzyme-immobilization matrix that maintains GOx activity, enhances substrate diffusion. The synergistic integration of these components yields high sensitivity, low detection limit, good stability, and strong selectivity, demonstrating strong potential for practical glucose monitoring. Future work will focus on assessing long-term stability in physiological media, validating performance with real biological samples, and exploring integration with miniaturized or wearable platforms for point-of-care applications. Additionally, strategies such as optimizing layer thickness, porosity, and enzyme loading will be considered to address the current limitation in the linear detection range and further broaden the sensor’s applicability in physiological environments.

## Data Availability

The data presented in this study are available on reasonable request from the corresponding authors.

## Abbreviations

The following abbreviations are used in this manuscript:

MWCNTs	Multi-walled carbon nanotubes
2-MIM	2-Methylimidazole
BSA	Bovine serum albumin
GOx	glucose oxidase
HCl	hydrochloric acid
CS	chitosan
FeCl_3_	Ferric chloride
NaCl	sodium chloride
KCl	potassium chloride
NaH_2_PO_4_	sodium dihydrogen phosphate
Na_2_HPO_4_	disodium hydrogen phosphate
K_3_[Fe(CN)_6_]	potassium ferricyanide
UA	uric acid
LA	lactic acid
AA	ascorbic acid
SPCEs	Screen-printed electrodes
SEM	scanning electron microscope
FT-IR	Fourier transform infrared spectrometer
XPS	X-ray photoelectron spectrometer
XRD	X-ray diffractometer
MOFs	Metal–organic frameworks
ZIF-8	Zeolite imidazolate framework-8
HRP	horseradish peroxidase
PBS	phosphate-buffered saline
